# Bridging Experimentation and Computation: OMSP for Advanced Acrylate Characterization and Digital Photoresin Design in Vat Photopolymerization

**DOI:** 10.3390/polym17020203

**Published:** 2025-01-15

**Authors:** Leah Okoruwa, Fatih Tarak, Farzaneh Sameni, Ehsan Sabet

**Affiliations:** 1 Additive Manufacturing Centre of Excellence Ltd., Derby DE23 8YH, UK or l.okoruwa@lboro.ac.uk (L.O.); f.sameni@am-coe.com (F.S.); 2 Wolfson School of Mechanical, Electrical and Manufacturing Engineering, Loughborough University, Loughborough LE11 3TU, UK; f.tarak@lboro.ac.uk

**Keywords:** vat photopolymerization, acrylates, binder development, photoresin, computerization, digitalization, mixing model, OMSP

## Abstract

Vat photopolymerization (VPP) is an additive manufacturing method that requires the design of photocurable resins to act as feedstock and binder for the printing of parts, both monolithic and composite. The design of a suitable photoresin is costly and time-consuming. The development of one formulation requires the consumption of kilograms of costly materials, weeks of printing and performance testing, as well as the need to have developers with the expertise and knowledge of the materials used, making the development process cost thousands. This paper presents a new characterization methodology for acrylates that allows for the computerization of the photoresin formulation development process, reducing the timescale to less than a week. Okoruwa Maximum Saturation Potential (OMSP) is a methodology that uses attenuated total reflection (ATR-FTIR) to study the functional group of acrylates, assigning numerical outputs to characterize monomers, oligomers and formulations, allowing for more precise distinguishment between materials. It utilizes the principles of Gaussian normal distribution for the storage, recall, and computerization of acrylate data and formulation design without the need to database numerous files of spectral data to an average coefficient of determination (R^2^) of 0.97. The same characterization method can be used to define the potential reactivity of acrylate formulations without knowing the formulation components, something not possible when using properties such as functionality. This allows for modifications to be made to unknown formulations without prior knowledge of their contents. Validation studies were performed to define the boundaries of the operation of OMSP and assess the methodology’s reliability as a characterization tool. OMSP can confidently detect changes caused by the presence of various acrylates made to the photoresin system and distinguish between acrylates of the same viscosity and functionality. OMSP can compare digitally mixed formulations to physically mixed formulations and provides a high degree of accuracy (R^2^ of 0.9406 to 0.9964), highlighting the future potential for building foundations for artificial intelligence in VPP; the streamlining of photoresin formulation design; and transforming the way acrylates are characterized, selected, and used.

## 1. Introduction

VPP is an additive manufacturing method that produces the highest surface quality and can create intricate designs and complex geometries [1]. VPP requires a photocurable resin that can be cured layer by layer to produce the desired geometry [2]. It is also possible to use VPP to produce composite materials, where solid particles are loaded into the photoresin binder and entrapped in the crosslinked matrix when the binder cures [3]. In many instances, the photocurable resin is developed specifically for the application [4], whilst in other studies, a commercially available resin with minimal knowledge of the properties is used [5]. Some studies have been conducted to develop photocurable photoresins and study the impact of particular resin components on resulting application properties. In a study by Qian et al. [6], a photoresin study was performed to analyze the volume shrinkage and conversion for alumina composite applications using ethoxylated pentaerythritol tetraacrylate (PPTTA), 1,6-hexanediol diacrylate (HDDA), and commercial photocurable resin. Real-time Fourier transform infrared spectroscopy (FTIR) was used to measure the conversion of C=C to C-C, and the photoresin was exposed to UV light. This study’s concentration of acrylate bonds, resin viscosity, and formulation conversion rates impacted the volume shrinkage. Another study by Basar et al. [7] conducted a binder study using an aliphatic urethane acrylate oligomer and a series of diluents and crosslinking agents. The rheology, cure depth, and mechanical and thermal properties of the formulations were then tested. When testing the impact of increasing oligomer content, there was an observation of high polymerization leading to embrittlement of the part, working against the objective of printing. Once the oligomer amount was optimized, the second stage of the study involved the use of N-vinyl-2-pyrrolidone (NVP) and HDDA to drop viscosity and improve molecule mobility for conversion, which was found to be true from the study’s findings. The authors also concluded that a drop in the abundance of high-functionality components has a negative impact on polymerization, an observation made in a number of other studies [8,9,10,11].

Developing and carrying out binder studies is very time-consuming and sometimes expensive to do. In many instances, a variety of acrylates must be obtained and mixed in a number of various quantities. Large enough samples of each binder, often >100 g samples for each formulation, must be made to meet the minimum vat tray volume to 3D print test specimens for formulation performance analysis. The analysis stage often requires a number of expensive pieces of equipment and considerable expertise to carry out experimentation properly. Performing tests involves the purchase/subscription of software and standards to ensure that the tests are being carried out and analyzed correctly. Various monomers and oligomers are available for purchase from suppliers and distributors. They come with technical data sheets to allow for more informed decisions to be made for formulation component selection. Characterizing properties often include viscosity, an indicator of diluent properties [12]; loading potential [13] and polymerization potential [7]; acid value; molecular weight, an indicator of the complexity and size of each molecule [14]; transition glass temperature, which shows the temperature application potential of material [15]; and lastly, functionality which shows the number of acrylates on the molecule of interest. Functionality is a useful parameter, showing the crosslinking potential and, to some degree, the mechanical properties that can be achieved when a particular monomer or oligomer is added to a formulation [16]. However, this value does not factor in key unique characteristics in each molecule that significantly contribute to their performance, allowing for minimal differentiation between molecules with the same functionality.

Additionally, when it comes to oligomers and bespoke reactive diluents, many photoresin manufacturers do not disclose other characteristics, such as molecular weight, as a way to protect their stoichiometric designs. Therefore, some assessments vital to oligomer suitability for a formulation cannot be made, such as determining whether an oligomer has a high viscosity due to high-density intermolecular forces developing due to additional polar functional groups that can lead to higher mechanical properties when used in a photoresin formulation or whether the high functionality is due to the bulkiness of the branching chains of the carbon backbone. There is currently no unique data characteristic that can provide such information.

Attenuated total reflection (ATR) is an analytical tool looking into compounds’ bond vibrations [17]. It is used as a tool to identify functional groups and characterize materials. The energy state of the functional group being analyzed is influenced by the bond type, its vibration type, and the atoms being covalently bonded to each other. The functional group will absorb a specific wavelength of light within the infrared spectrum, allowing it to reach an energized state. This wavenumber that it absorbs is where the peak appears on the ATR spectrum [18]. The convenience of ATR is that there is no need for special sample preparation methods for traditional methods of FTIR [19]. The peak at 810 cm^−1^ is used to identify the alkene bond situated on an acrylate. It has been used in the literature previously to obtain information on the reaction behavior of photoresins [20,21].

In this study, a code was developed to analyze the peak found at 810 cm^−1^ directly from the output data of an ATR instrument. The outputs were designed to assign a set of characteristics that were believed to be more informative than functionality. While functionality is limited to acrylate monomers and oligomers, the developed characterization methodology and outputs can also be applied to acrylate formulation, providing further differentiation between similar acrylate molecules and place as a tool to aid the streamlining of binder formulation studies without photoresin developers having to divulge sensitive information about their bespoke acrylates. In this study, the bounds of operation for this code were determined, and a series of validation tests were performed to assess and present the potential of this new tool. Along with the code outputs, rheology, tensile properties, cure depth/thickness, polymerization conversion, and hardness were measured and reported for green body specimens.

## 2. Materials and Methods

Photoresin formulations were created using acrylate materials (Rahn AG, Zurich, Switzerland) Table 1. The formulations created can be seen in Table 2. The formulation components were mixed in a DAC 150FVZ-K speed mixer (Hauschild, Hamm, Germany) at 2000 rpm for 5 min. In formulations intended for curing, Phenylbis(2,4,6-trimethyl benzoyl)phosphine oxide (BAPO) photoinitiator (Rahn AG, Zurich, Switzerland) was added to the speedmixed acrylate formulation and sonicated in a Clifton SW6H sonicator (Nickel-Electro, Weston-super-Mare, UK) for 30 min to encourage the dissolution of the photoinitiator [22]. Following this, the photoresin was speed-mixed at 2000 rpm for an additional 5 min to ensure the homogenous distribution of the dissolved photoinitiator. The formulations were placed in a Photocentric Cure L2 (Photocentric, Peterborough, UK), using the convection oven functionality to set the photoresin temperature to 25 °C.

### 2.1. Rheology

Rheology studies were performed on the formulations of photoresins and slurries. The viscosity of samples was measured on a Brookfield RST-CPS-P rheometer (Ametek, Braunstone Town, UK). Approximately 0.2 g of photoresin was deposited on the plate of the rheometer, and the rheology study was performed at 25 °C at a constant shear rate of 800 s^−1^. The viscosity was averaged across 120 data points. For slurries, the tests were also run at 25 °C, but the shear rate was increased from stationary to 200 s^−1^ to collect 60 data points for the upwards shear and downwards shear behavior for the suspension.

### 2.2. Ultraviolet Light (UV) Exposure

An Anycubic photon mono 4K printer (Shenzhen, China) was used to carry out UV-exposure-related operations. Cure disks of 10 mm diameter and 100 µm thickness were designed using computer-aided design (CAD) software. Each formulation was poured into a vat tray and exposed to 4000 μW/cm^2^ of 405 nm UV energy for 10 s. Each formulation was exposed to the same curing conditions to study the extent of conversion attainable for each mixture, therefore assessing the reactivity of the mixtures formulated, regardless of whether full conversion was attained. The disks were removed from the vat tray’s base and cleaned with isopropyl alcohol (IPA). A micrometer was used to measure the cure thickness. The same methodology was used to prepare cure disks for ATR-FTIR to measure the polymerization conversion.

### 2.3. Mechanical Testing

**Figure 1:**
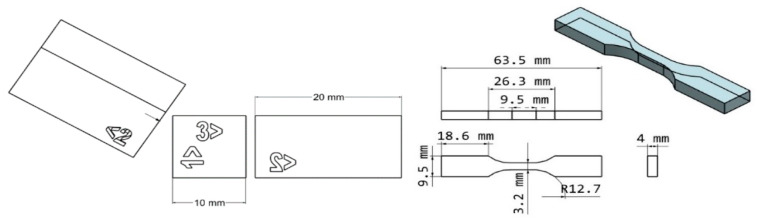
Geometry of test specimens. (**Left**) hardness bars; (**Right)** type V tensile bars.

ASTM D638 Type V dogbones were printed for tensile testing [23], and a Multitest- dV(u) (Mechmesin, West Sussex, UK) was used to perform tensile tests at 1 mm/min in line with ASTM D638 instruction (Figure 1, right) [23]. Shore D hardness testing was performed on specimens following dimension guidelines in ASTM D2240 (Figure 1, left) [24]. An average of the center point measurements was taken from seven samples.

### 2.4. ATR-FTIR

A Bruker Alpha Platinum-ATR (Bruker, Billerica, MA, USA) was used to perform ATR-FTIR analysis on uncured and cured samples of photoresin with 40 scans at a nominal resolution of 2 cm^−1^, as this was the highest resolution attainable for this instrument. Before the testing commenced, the ATR crystal was cleaned with IPA and allowed to dry fully. A background measurement of the atmosphere was taken to minimize the corruption of results. Once the background test was completed, <50 mg samples (1 drop) of material were deposited onto the surface of the ATR crystal for uncured samples. For cured samples, the cured disk was placed on the surface of the ATR crystal using cleaned tweezers to prevent sample contamination. The anvil was then lowered to show the red indicator, centered in its window, indicating ideal sample contact with the crystal. The test was run on OPUS, allowing for the preview of the ATR-FTIR spectrum. This preview was then used to assess the level of noise and anticipate the quality of the test. When the quality was visually ideal for analysis, the ATR-FTIR run commenced, and the test was conducted without interference.

**Figure 2:**
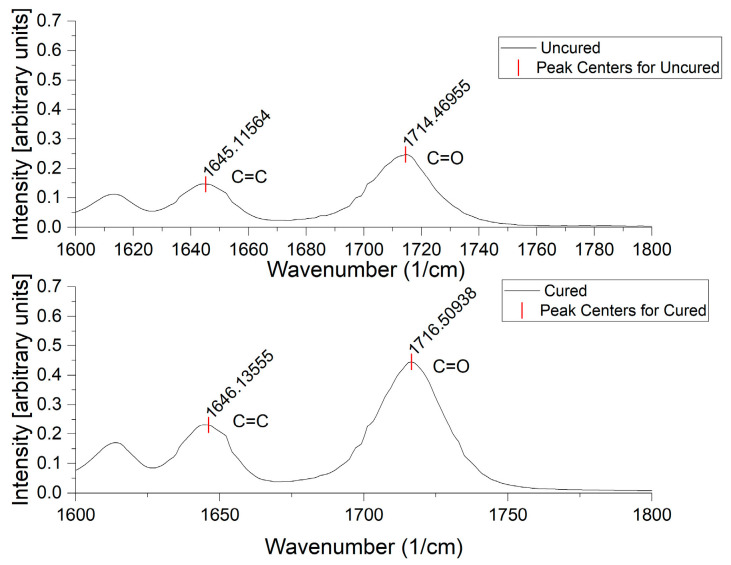
Graph of the ATR-FTIR raw data for an uncured and cured sample of the same formulation, locating the peaks of interest used to determine conversion calculations in this study.

For conversion (ξ) calculations (Equation (1)), the peak area (*A*) of the C=C (alkene stretching) appearing at 1635 cm^−1^ on the spectrum was compared before (U) and after (P) exposure to UV light. The carbonyl vibration appearing at 1720 cm^−1^ (Figure 2) was used as a baseline to account for variations in sample size [25].

(1) $$\xi =1-\left[\frac{{\left(\frac{A\left(C=C\right)}{A\left(C=O\right)}\right)}_{P}}{{\left(\frac{A\left(C=C\right)}{A\left(C=O\right)}\right)}_{U}}\right]$$

### 2.5. OMSP Code Development

The code was designed to allow the upload of multiple files in a single run in the file formats of .dpt, .xls, .xlsx, and .txt. The alkene 810 cm^−1^ and the carbonyl 1720 cm^−1^ peaks were then identified. Independent linear baseline subtractions are performed for both peaks, and then Gaussian, Lorentzian, and Linear Combination Pseudo Voigt methods [26] are run on the peak to find the best fit. The best fit for each peak was determined using the Akaike Information Criterion (AIC) [27] and root mean square error (RMSE) [28,29], with AIC being the main deciding parameter for the best-fit type. Peak integration was then used to find the areas under the peaks. Peak normalization was performed using the carbonyl peak to correct the peak data, accounting for variations in sample size and intensities that may skew the results [30].

Table 3 shows the output when the OMSP code is run for individual acrylates and formulations.

For the development of the code for individual acrylate formulations, a “home” wavenumber was determined through the assessment of the simplest molecular form of different functionality acrylates. The area of the alkene peaks was corrected and limited to the density of alkene bonds that absorb wavenumbers equal to or greater than the home for the functionality it belongs. This adjusted area was then normalized with the C=O peak to provide an acrylate OMSP value.

## 3. Results and Discussion

### 3.1. Boundaries of Operation for OMSP

The first stage of the study was to establish the operational boundaries of OMSP. Given that ATR-FTIR was used for analysis, focusing on a specific peak, the compatibility of this method with the broad range of reactive materials used for VPP needed to be established before its uses and benefits could be clearly defined.

**Figure 3:**
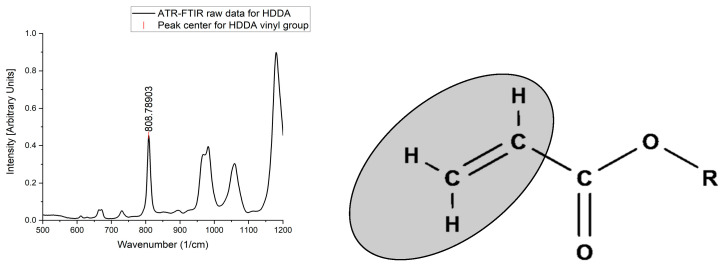
(**Left**): ATR-FTIR raw data highlighting the vinyl group peak of interest for OMSP. (**Right**): Displayed formula of an acrylate functional group, highlighting the bond used to characterize acrylates in OMSP.

The 810 cm^−1^ peak is specific to the bending out-of-plane vibration of the vinyl groups situated adjacent to the double bond of an acrylate functional group Figure 3. This peak can be found in the unique fingerprint region of ATR-FTIR spectral data [31], meaning it can be easily influenced by surrounding electron density from nearby functional groups such as α-methyl groups on methacrylates. As a result, the modified acrylates, such as methacrylates and other non-acrylate photopolymerizable materials, vibrate at a different wavenumber to a standard acrylate, creating their own OMSP scales. As a result, when methacrylates are included in OMSP characterization alongside acrylates, the materials appear more reactive than they truly are, providing a false OMSP result). Photopolymerizable non-acrylate materials such as NVP appear as voids as they are not acrylates that vibrate in the spectral point OMSP uses.

However, when OMSP is used for acrylate materials, OMSP is able to distinguish between acrylate materials with high accuracy, which is not possible with the use of functionality and viscosity, meaning they would have to have been physically tested to distinguish their differences in the absence of OMSP. The outputs obtained for the distinction made between similar monomers agreed with mechanical results and degree of cure results. Additionally, the outputs of OMSP were able to be used to create a database of acrylates, allowing for formulations to be mixed computationally and showed high similarity to the result of physically mixed systems, with an average R^2^ of 0.9685 This showed the precise accuracy of OMSP and the potential for OMSP to be used to streamline the photoresin design process and reduce the need to acquire numerous acrylates to mix and test numerous formulation.

### 3.2. Determination of Functionality Home

**Figure 4:**
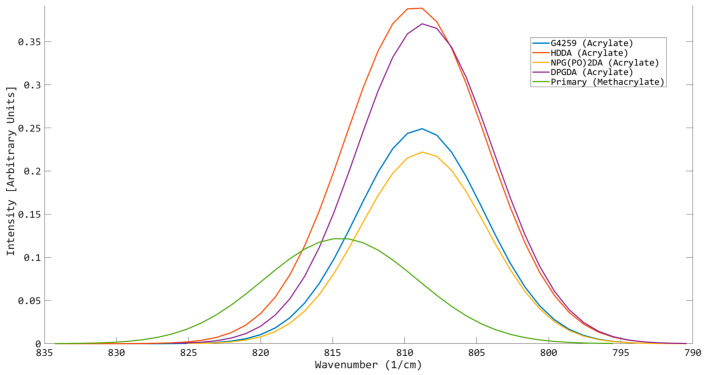
OMSP output for a selection of difunctional oligomers and monomers.

A series of difunctional acrylates were tested to observe where their peak positions resided (Figure 4). With the same functionality and additional branching groups on the carbon backbone, it was expected that little to no spectral shift would be seen, confirming a key parameter in this method: functionality home. For the oligomers included in this test (G-4259 and Primary), any change in the peak position was to be an indication of additional functional groups in close proximity to the acrylate alkene, influencing the energy state of the alkene bond and altering its reactivity [32]. As seen from the results, each of the difunctional reactive diluents peaked at similar wavenumbers as well as G-4259, making 808.789 cm^−1^ the home wavenumber for difunctional acrylates. However, the oligomer, Primary, experienced a spectral shift from the home position of 808.789 cm^−1^ to 814.908 cm^−1^, suggesting Primary to be a more reactive oligomer; however, this is not the case, as Primary is a methacrylate.

In other studies using ATR-FTIR to assess various chemical mixtures, authors have found intermolecular forces to influence their systems in certain mix ratios, leading to peaks shifting to higher wavenumbers [33].

**Figure 5:**
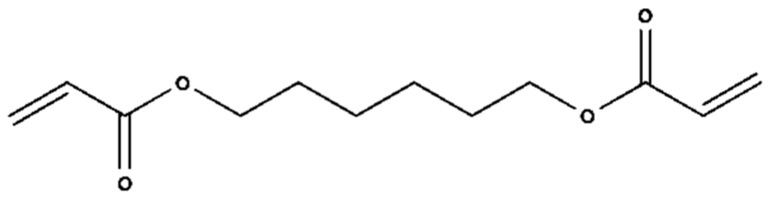
Displayed diagram of HDDA.

The findings of this led to the addition of “functionality home” to the analysis method of OMSP, the foundation to the analysis of formulation and the assignment of “pseudo-functionality” to mixtures of acrylate material, which was not previously possible. Functionality home takes the simplest structure of each functionality acrylate and uses it as a benchmark of the expected peak position of that functionality, creating a scale. For instance, HDDA (Figure 5) was taken as the functionality home molecule for difunctional acrylates as it has no stereoregularity or additional functional groups that could alter the reactive state of the acrylate group’s alkene bond. Additionally, the two functional groups of HDDA are situated on the extremities of the chain, assuming to have minimal influence on either acrylate group.

Table 4 shows the molecules selected as the functionality home for each functionality. Pentaerythritol tetraacrylate and dipentaerythritol pentaacrylate were unavailable for physical testing, and therefore, interpolation was used to determine their peak positions.

**Figure 6:**
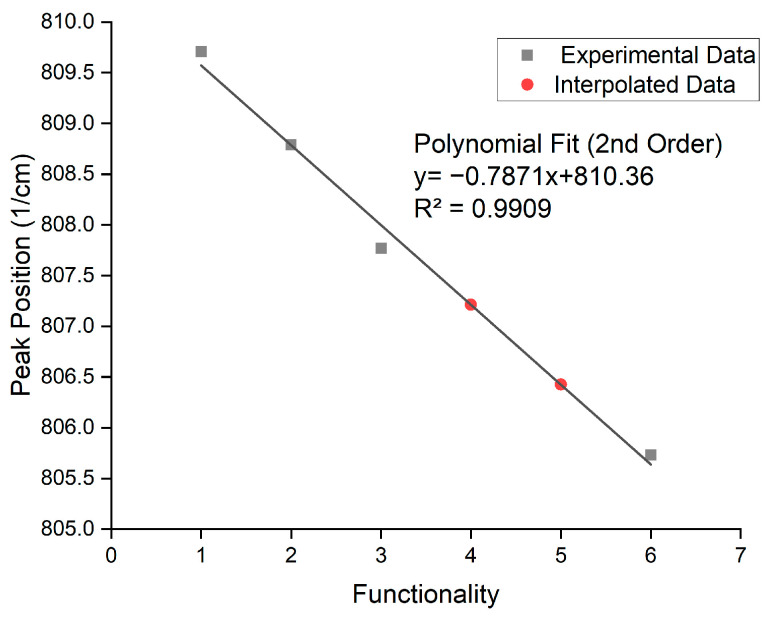
Output peak positions and interpolated positions for tetra- and penta-functional acrylates based on the code using the original curve identification method.

**Figure 7:**
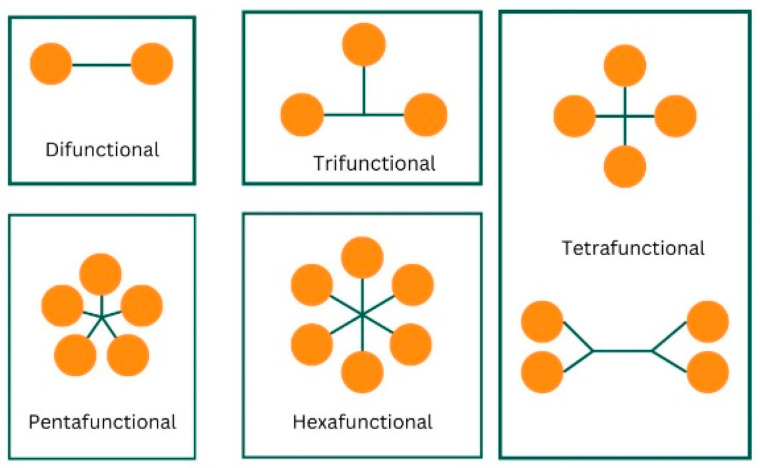
Ball and stick illustration of different functionality acrylates, showing the positioning proximity of functional groups to each other on a molecule.

When the peak positions of each functionality home were plotted on an axis, the trend seen in Figure 6 was expected. As the functionality increased, there was an observed decrease in the peak position. This gradual shift in the peak position with increasing functionality was linked to a decrease in the mean reactive state of each acrylate as its functionality increased. This shift to a lower wavenumber is an indicator of increased stability [34]. This stability is due to conjugated π bond systems being created between the carbonyl and alkene bonds within the acrylate, leading to delocalization [35,36]. The increase in functionality leads to an increase in proximity (Figure 7) between zones of electron delocalization, allowing for delocalization to spread across the entire molecule, stabilizing it [36].

There is a steady decrease in the reactive state of the acrylate as the functionality increases, fitting a linear fit. The significance of this finding is that it completely changes the way in which reactivity is described in photopolymerization and additionally allows for any acrylate mixture to be assessed on its overall reactive state and linked to its proximity to a functionality home—pseudo-functionality—regardless of whether there are additives such as dispersants of solid fillers.

Figure 6 shows the functionality home for the mono, di, tri, and hexafunctional home molecules. As seen from the results, there is a gradual shift of the peak to a lower wavenumber as the functionality of the acrylate increases. As the functionality of the structure increases, there is an observed spectral shift.

**Figure 8:**
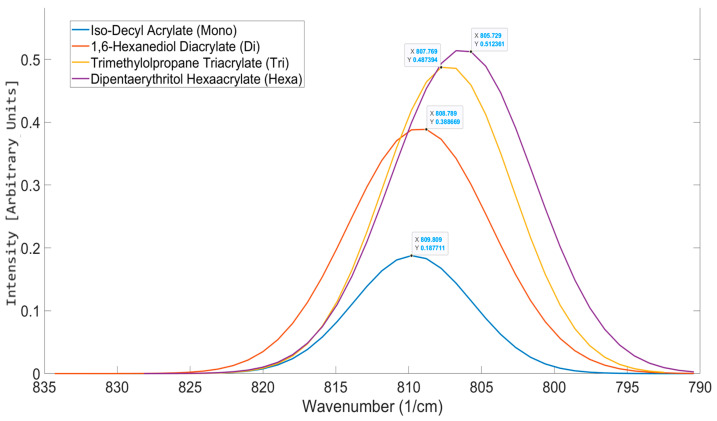
OMSP outputs for the selected acrylate monomers used to determine the functionality home peak positions for the OMSP code.

Before the development of functionality home, the functionality itself was used as a measure of an acrylate’s reactivity. In the development of formulation, higher functionality was associated with the development of a higher degree of crosslinking but never distinguished from the reactive state of the high functionality acrylate. A study by Van der Voorde [37] concluded that the functionality of an acrylate material could not be correlated to its reactive state; however, this study assumed that there was no difference between the reactive state of an acrylate functional group situated on a difunctional acrylate and an acrylate on a tetrafunctional acrylate, as illustrated in Figure 8.

Additionally, with increasing functionality, there appears to be a decrease in the aspect ratio of the peak (Table 5). A study by Torii et al. [38] looked into the impact of electron delocalization on the peak intensity of infrared spectroscopy. Their study linked the delocalization of the electron to the charge flux, showing that with greater electron delocalization, there is a large change in the dipole moment, causing a more intense peak in the infrared spectrum—as seen in the OMSP peaks—with increasing functionality.

#### Trends in Peak Positions When Different Functionalities Are Mixed

**Figure 9:**
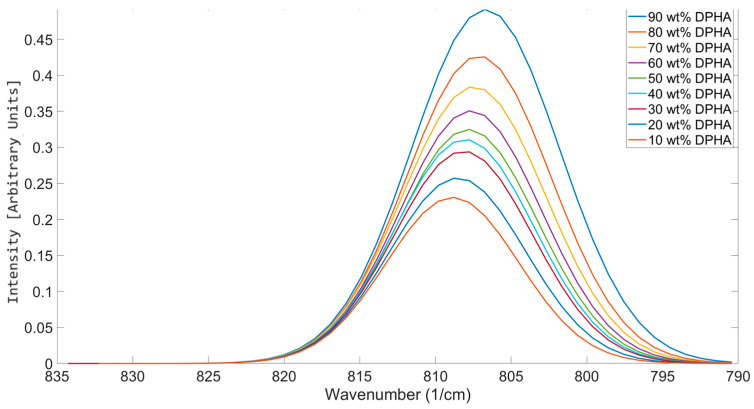
Graphical OMSP output for varying balances of IDA (monofunctional) and DPHA (hexafunctional).

Variations in reactivity with acrylate functionality have been observed in the literature [39,40]. IDA and DPHA were mixed at varying concentrations to study the impact of mixing different functionality components on the OMSP area and peak position (Figure 9). The results showed a gradual increase in the peak position with increasing amounts of IDA, supporting the trend seen in Figure 6. As per the results seen in Figure 6 and 8, an increase in the functionality of the acrylate leads to a decrease in the peak position. Table 5 also showed that with the increase in functionality, an increase in alkene bond abundance is also observed. The results shown in Figure 8 support the observations made in these previous figures. The impact of increasing the content of DPHA led to an increase in the peak area, while the higher stability of DPHA compared to IDA is reflected in the gradual decrease in the mixture peak position. The code’s ability to detect the subsequent changes to the peak position provides the opportunity to assign a pseudo-functionality to formulations, relating the mean reactive state (peak position) of the formulation to one of the functionalities outlined in Table 5.

### 3.3. Comparison Between Acrylates and Methacrylates

The spectral shift observed by the aliphatic urethane difunctional methacrylate oligomer indicated that the oligomer was significantly more reactive compared to the other tested difunctional oligomer, G4259, an aliphatic urethane acrylate. In the literature, methacrylates and acrylates are interchangeably used in formulation development and, in many cases, mixed to form formulations for photoresins [41]. However, the methyl group situated on the acrylate functional groups causes physical obstruction to the photoinitiator during the polymerization process, often referring to them as less reactive than their acrylate counterpart [42]. In a study by Maruo et al. [43], TMPTA and TMPTMA were compared. The conversion achieved for both acrylates was analyzed at 20 wt% and 40 wt%, showing the TMPTMA to show a lower conversion to TMPTA. When analyzing these two structures using OMSP, the output showed a spectral shift to a higher wavenumber, as seen by the difunctional methacrylate oligomer. Based on this shift, the code determined the pseudo-functionality of TMPTMA to be monofunctional, making it appear to be a highly reactive trifunctional acrylate, which is not the case seen in Maruo’s study.

**Figure 10:**
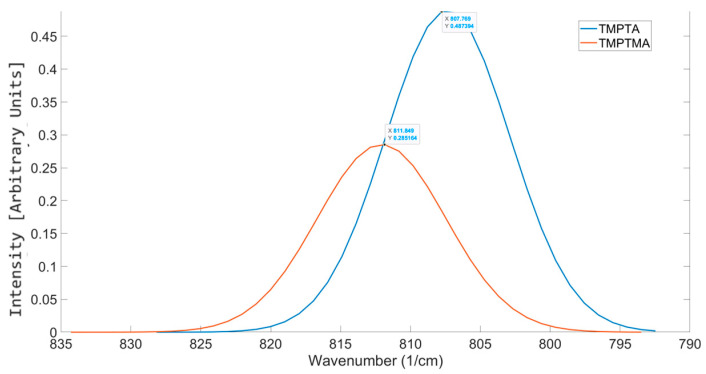
Comparison of OMSP between TMPTA and TMPTMA.

Formulations M.1 and M.2 were made to compare the mechanical properties of TMPTA and TMPTMA (Figure 10) to the results seen in OMSP output and Maruo’s study. The OMSP output for TMPTMA had a higher OMSP area of 0.1792 compared to the TMPTA formulation, which had a lower OMSP of 0.1663, showing TMPTA to have a high potential for saturation with its increased density of polymerizable alkenes. The peak positions showed the TMPTA formulation to peak at a lower wavenumber of 807.54 cm^−1^, whilst TMPTMA peaked at 807.68 cm^−1^. Although similar in peak position, the 20 wt% abundance was enough to show a resultant shift in the peak position of the formulations. However, the conversion measurement for TMPTA showed 49.04%, whilst TMPTMA had a conversion of only 29.7%. This lower reactivity has been reported in previous literature [44], where the steric hindrance caused by the α-methyl groups of methacrylates reduces the access photoinitiators have with the adjacent alkene bond, as well as restricted mobility due to increased viscosities as seen in Table 6, where higher viscosities were observed in M.2 and M.4. The mechanical properties also showed TMPTA to be the stronger material, with a UTS of 44.18 MPa and Young’s modulus of 462.8, whilst TMPTMA’s values were 31.25 MPa and 336.71, respectively. Similar outcomes were seen when IBOA was compared to IBOMA.

**Figure 11:**
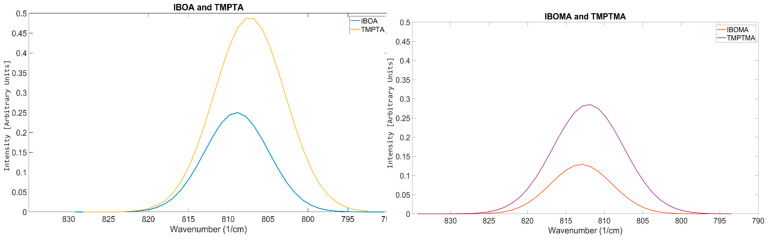
Similarities in OMSP peaks seen in acrylate monomers, also seen in methacrylate monomers.

A notable difference in the results found for acrylates and their methacrylate counterpart is the aspect ratio. Acrylates appear to have a lower aspect ratio compared to the methacrylate alternative. Not only does the methacrylate shift to a higher wavenumber, but the spread of reactivity also appears greater. Despite the tests showing that methacrylates appear to vibrate at a higher wavenumber than their acrylate counterpart, the relationship between functionality and peak position IBOA and TMPTA can also be seen in IBOMA and TMPTMA (Figure 11). Therefore, OMSP at this stage excluded methacrylate components from the formulations tested.

### 3.4. Detection of Non-Acrylates

**Figure 12:**
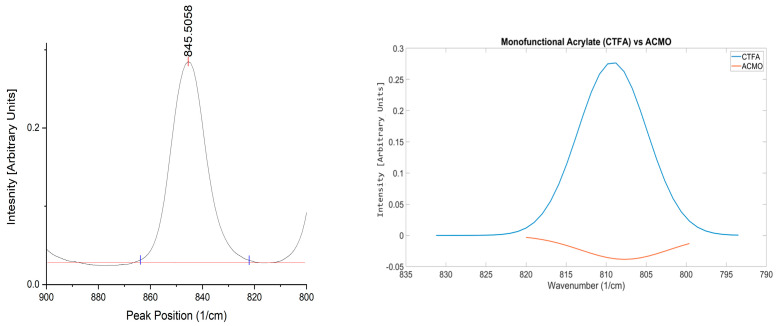
(**Left**): Shifted peak position of ACMO’s vinyl group appearing at a higher wavenumber due to the effects of ACMO’s acrylamide group. (**Right**): OMSP outputs of CTFA and ACMO. Display of AMCO and OMSP code incompatibility.

ACMO is a reactive diluent used in formulations to provide stiffness and lower the viscosity of formulations [45]. As seen in Figure 12(right), the OMSP area generated for ACMO shows a negative region of absorbance intensity despite the peak still appearing within the acrylate region. The reason for the appearance of ACMO in Figure 12 is due to the acrylamide group’s electron-donating effects causing the vinyl group in ACMO’s structure to appear at a higher wavenumber and out of the range used for OMSP, as shown in Figure 12(left). Based on the appearance of the peak in the figure, it is believed that the presence of ACMO would present as a void in formulation OMSP, making formulations appear to have lower conversion potential than they truly do. The void in the OMSP area was studied and compared to a monofunctional acrylate reactive diluent, CTFA, when both diluents were added to G-4259.

**Figure 13:**
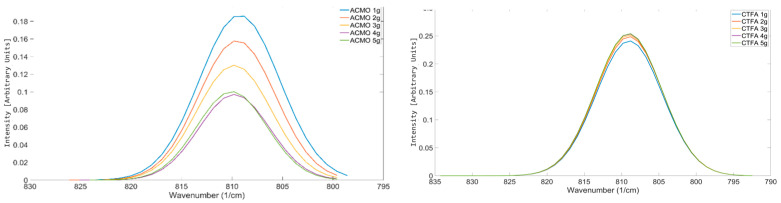
Comparison of how various amounts of monofunctional diluent impact the characteristics of the mixture’s OMSP curve. (**Left**) ACMO; (**Right**) CTFA.

Figure 13 shows the anticipated results. As there was an increase in the abundance of ACMO, there was also an increased presence of voids in the acrylate sample measurements, leading to a decrease in the OMSP area.

**Figure 14:**
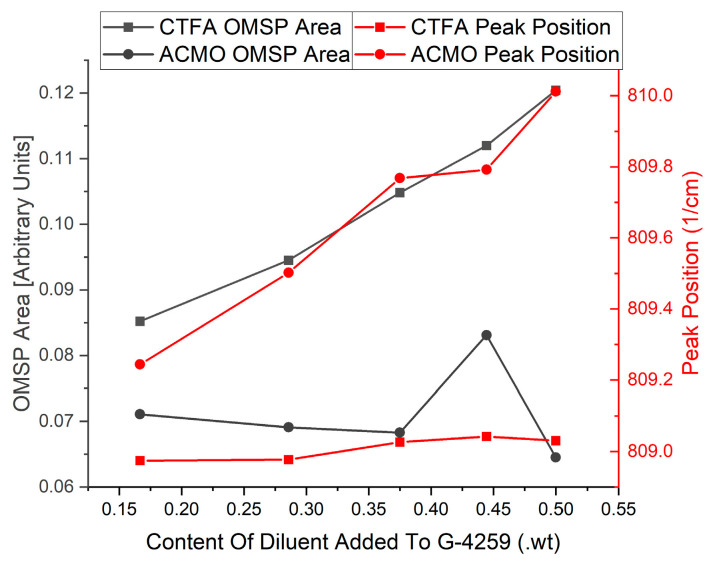
The effect on OMSP area and peak position when an acrylate detected by the OMSP code (CTFA) is added to G-4259 compared to when ACMO is added to G-4259 (undetectable with the code’s current version).

However, CTFA shows different results in Figure 13, where the OMSP area increased with increasing monofunctional acrylate abundance. Although ACMO is photo-polymerizable, the structure causes shifts in the OMSP area due to the morpholine group [46], making it undetectable under OMSP analysis. The significantly smaller molecular structure of CTFA compared to G-4259, whose molecular weight is primarily linked to the complexity of intermediate branching of the molecule, as the component is only difunctional [47]. The peak position was also influenced by the increasing presence of monofunctional diluent in both ACMO and CTFA samples. Therefore, OMSP was able to detect the presence of ACMO in the system due to the monofunctional diluent’s peak shift influence, as seen in CFTA (Figure 14). However, the OMSP outputs of ACMO-containing formulations could not be linked to their potential for crosslinking.

The analysis conducted was plotted to compare the trend seen in the addition of CTFA and ACMO to G-4259. As seen from the result, the steady expected changes are clear and consistent with the addition of CTFA to G-4259; however, when ACMO was added, there were more observed fluctuations, refining the current scope of OMSP to solely acrylate compounds.

### 3.5. Application of OMSP on Acrylate Systems

From this stage of the study, OMSP was used on various acrylate monomers and oligomers to demonstrate the efficacy of the characterization method. A series of tests were conducted to show how OMSP could be used to obtain acrylate properties and compare the observed trends found in the results of OMSP outputs to experimental findings via conversion testing and, in some cases, tensile testing.

OMSP being able to characterize acrylates by their potential reactivity and create unique Gaussian curves for them allows formulations to be directly compared in the same way as their components, meaning easier adjustments can be made to the formulation. The graphical presentation of the acrylate in Figure 8, for instance, allows the effects of the addition of acrylate components on the shift in reactivity for the formulation mixture through changes in the position and aspect ratio of the curve. The numerical outputs, such as OMSP areas, allow you to compare potential reactivities to each other directly, whilst the aspect ratio indicates the spread of reactivity. Therefore, the simple outputs of OMSP can provide more information about an acrylate system to a much higher degree of detail than is currently available without the need for acrylate manufacturers to divulge sensitive information about the molecular composition, allowing the user to make comparisons and decisions in a quicker, less subjective, and confident way, whilst the manufacturers can protect their intellectual property.

#### 3.5.1. Differentiating Between Similar Reactive Diluents

Both DPGDA and NPG(PO)2DA are difunctional glycol diacrylates with viscosities of 15 mPa·s [48,49]. The difference between the groups is the connections in the carbon backbone, where NPG(PO)2DA contains an additional propane chain in the center of the backbone, whilst DPGDA connects the two acrylate functional groups with an oxygen atom. Despite both having the same viscosity when measured as a component, they resulted in different viscosities when mixed as formulations. This result of varying viscosity is due to variations in intermolecular interactions in the mixture caused by NPG(PO)2DA’s propoxylate group [50].

**Figure 15:**
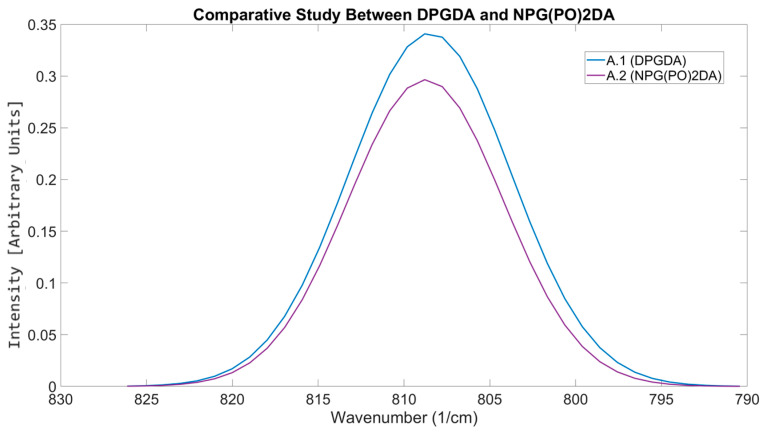
OMSP output of formulation containing DPGDA (A.1) compared to the output containing NPG(PO)2DA (A.2).

Figure 15 shows the OMSP output for the DPGDA, NPG(PO)2DA, formulation A.1, and formulation A.2. Table 7 shows the formulation viscosity, conversion, tensile properties, and hardness results of the two formulations. As seen from the results, both DPGDA and NPG(PO)2DA have very similar peak positions, a relationship which has translated to the formulations made from them. The formulation containing DPGDA had an OMSP area of 0.1435, whilst the NPG(PO)2DA formulation had a lower OMSP area of 0.1354. The additional branching of NPG(PO)2DA was detected as the cause of the drop, as the branching methyl groups increased the bulkiness of the acrylate, lowering the space available for the polymerizable alkene bonds [51]. Additionally, the conversion results support this, as the DPGDA formulation showed a conversion 21.08% greater than the NPG(PO)2DA-substituted formulation.

Although both difunctional diluents had the same theoretical viscosity, the rheology results showed the formulation containing NPG(PO)2DA to have a viscosity 97% greater than the one with DPGDA. As the peak positions for both formulations show minimal difference, the increase in viscosity for A.2 is linked to the branching of the NPG(PO)2DA methyl groups, adding to flow resistance [52]. The larger molecular weight of NPG(PO)2DA leads to a higher hydrodynamic volume [53,54]. These observations made due to the difference in molecular bulkiness are also reflected in the cure depth, with DPGDA having a cure depth 4.42% greater than that of NPG(PO)2DA. These differences identified by the OMSP code were present in the mechanical properties, as A.1 had a higher UTS (11.66% increase). The stiffness due to additional crosslinking seen in the conversion data is also shown in Young’s modulus (3.71% increase) and a 2.77% increase in the shore D hardness [55]. The strain at break, however, is similar between both formulations, with the difference in strain at break between the two only being 0.05%.

#### 3.5.2. Differentiation Between Oligomer Types

**Figure 16:**
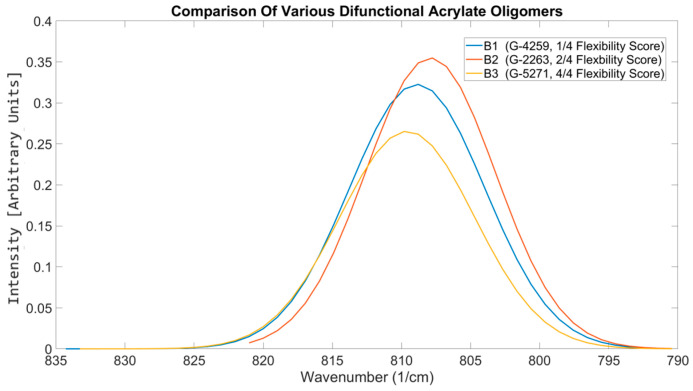
OMSP outputs comparison photoresins of the same reactive diluents but different flexibility oligomers.

Oligomers are typically high complexity and high molecular weight acrylates directly associated with the resultant mechanical characteristics the final photoresin would possess [56]. Due to their high viscosities, they are often not printable unless mixed with formulations with low viscous diluent materials [57]. The details of the oligomer beyond their anchor type are often not disclosed by the manufacturer, including the molecular weight. Therefore, the performance data provided by the manufacturer and the featured details on the technical datasheet are relied upon during the selection process. As the OMSP area gives a good indication of the bulkiness of the components, as seen in the differences between NPG(PO)2DA and DPGDA and the peak position can be used to understand how the functionality of the acrylate and additional functional groups impact the energy state of the formulation, OMSP was tested as a tool to understand and predict the mechanical performance of various oligomers. Therefore, three difunctional oligomers of varying structures and degrees of flexibility were compared. G-4259, the aliphatic urethane acrylate, was described by the manufacturer as low-flexibility (1/4) [58]; G-2263, an epoxy acrylate with a flexibility score of 2/4 [59]; and G-5271, a highly flexible (4/4) amine acrylate [60] were tested and mechanical properties compared to their OMSP outputs (Figure 16).

G-2263, having the highest OMSP area, also presented the highest conversion; however, G-5271 was expected to have the second-highest conversion based on the OMSP area, but the conversion was significantly lower. When assessing the mechanical properties of the various formulations, the mechanical properties of G-2263 were the highest, correlating with the high OMSP area and conversion [61]. However, according to Table 8, G-5271 showed significantly low mechanical strength and Young’s modulus. When analyzing the stiff oligomers, the OMSP was again able to correlate to the conversion and mechanical properties, but when assessing flexible components, the subsequent mechanical properties were not detectable. Amine acrylates utilize hydrogen bonding and their low conversion nature for flexibility [62]. Amine functional groups are not considered in the OMSP analysis, and therefore, variations in mechanical behavior could not be preempted. The aspect ratio of the various formulations, however, correlated with the properties, showing the broader B.3 curve to have inferior mechanical strength compared to narrower peak formulations. The poor tensile of the amine acrylate was expected, as similar performance had been reported by the manufacturers, which is assumed to be related to the unique design of their oligomer.

#### 3.5.3. Detection of Excess Diluent

**Figure 17:**
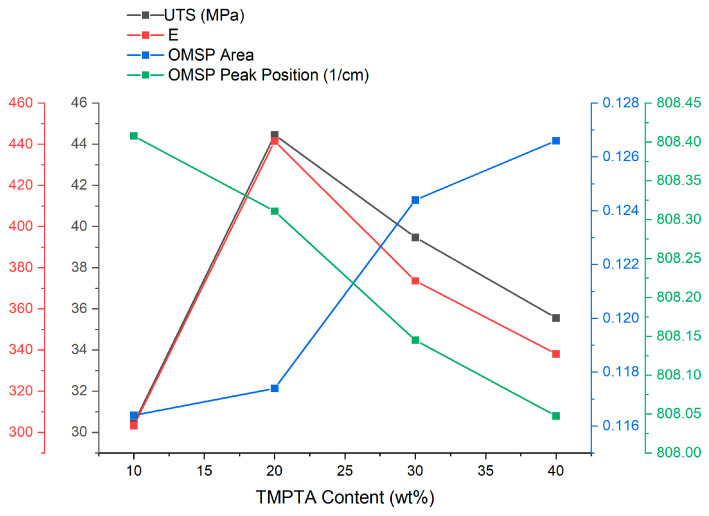
The effect of changing TMPTA content on the tensile UTS and Young’s modulus of the green body.

A challenge of photoresin formulation is the identification of excess materials [7]. A series of acrylate formulations were developed and tested with incrementally increasing amounts of TMPTA. The OMSP outputs were compared to the resultant formulation properties (Figure 17). With the increasing amounts of TMPTA, there was an observation of a gradual change in the peak position, resulting in the change of the formulation’s pseudo-functionality from a difunctional energy state to a trifunctional energy state. TMPTA has a higher functionality and lower molecular weight than PEG200DA, making it a component that provides a higher density of acrylate functional groups [37]. OMSP also reflects with increasing TMPTA content. The decrease in aspect ratio with an increase in functionality, seen when comparing various functionality home acrylates, can also be seen in this study, where increasing TMPTA content lowered the aspect ratio of the system.

The mechanical properties did not follow the same trends seen in the OMSP (Figure 15). The tensile results suggest the formulations were negatively impacted by excessive acrylate functional groups, resulting in a reduction of tensile performance in concentrations exceeding 20 wt% (Table 9). Despite the decline in mechanical properties, the lowest mechanical performance was still observed in the lowest TMPTA content. In a study by Pongwisuthiruchte et al. [63], the same observation was made, where an improvement of polymer properties was initially observed with the introduction of poly(ethylene glycol) dimethacrylate as a reactive diluent in their photocurable study. The authors reported a decline in performance when the diluent was in excess, believed to be due to TMPTA’s trifunctionality causing excessive crosslinking leading to the embrittlement of the polymer. The same effect can be seen in other studies [7,64,65]. As the oligomer used in the formulation of the D series was the only component mentioned by the supplier to provide good hardness and was also fixed in each formulation, there was little observed change in hardness values across the formulations.

#### 3.5.4. Detection of Non-Reactive Additives

In addition to acrylates and photoinitiators that make up photoresin formulations [66], additional materials can be added to provide additional functionality to the printed composites [67]. Particle agglomeration is a challenge encountered in heterogenous photoresin systems [68]. Dispersants have been used in the literature to help better disperse solids in the formulation [5,69,70]. In dispersant studies, dimensional accuracy has been one of the characterization methods that aid in the optimization of the slurry formulation [71]. Although dimensional accuracy has been linked to shrinkage as a result of polymerization [72], there has not been a study into how the dispersant added to the photoresin impacts the reactive state of the photoresin phase. Different dispersants have varying acid values [73]. With the increase in system acidity, the reactivity of the photoresin system was expected to increase [74].

DISPERBYK-111 (BYK-Chemie GmbH, Wesel, Germany) is a polymeric wetting and dispersing additive used in additive manufacturing applications [75]. This dispersant was added to the photoresin formulation in different quantities. The void caused by the dispersant was studied using the OMSP area, and changes in reactivity were studied with the peak position and validated with ATR conversion (Table 10). The results of this study show that despite the dispersant reducing the abundance of acrylate functional groups, there was a shift in the peak position to a more reactive energy state with increasing dispersant content, along with a gradual increase in the conversion of the formulation. The presence of dispersant causes this increase in reactivity and plays a contributing factor to dimensional accuracy challenges in VPP [76].

### 3.6. Simulation of Photoresin Formulations

In the development of the OMSP principle code, datasets of acrylate formulations and components were fitted to Gaussian and Lorentzian models, and the AIC was used to compare the peak shape. It was found that the curve of interest for OMSP adopted a Gaussian fit type, allowing for the development of the computerized mixing model. The Gaussian mixing model is a methodology that combines a series of Gaussian plots through a summation methodology [77].

**Figure 18:**
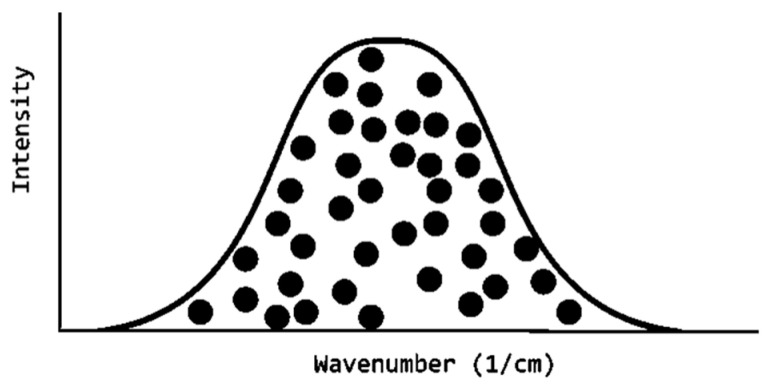
Illustration of how OMSP analyzes formulations. Each ball represents an alkene bond vibrating at its energy state, taking up the shape of a normal distribution (Gaussian curve).

The idea behind the use of the Gaussian mixing model was based on the hypothesis that an acrylate system can be statistically seen as a general population of acrylate molecules vibrating at their frequency (within the typical range for the ATR-FTIR peak of interest). Naturally, the population distributes across the ATR-FTIR peak position range in a normal distribution (Figure 18). This is true as all results run on the OMSP code showed a likeness to Gaussian fit.

**Figure 19:**
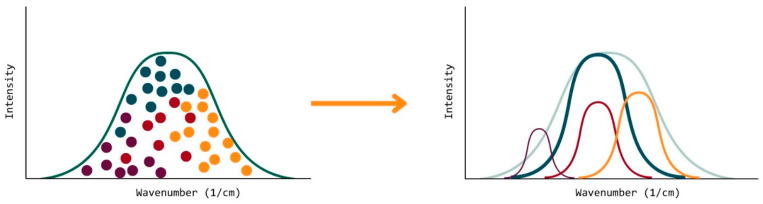
Two approaches of looking at a formulation OMSP curve. (**Left**) Looks at the system as clusters of energy states belonging to a particular component. (**Right**) Looks at each component as an individual Gaussian curve contained within the overall formulation Gaussian curve. Each color representing a different monomer/ oligomer used to produce the formulation studied.

However, when applying the Gaussian mixing model to this case, the formulation being analyzed by the OMSP code is, in fact, a series of clusters, where each cluster is an acrylate component used to make up the formulation (Figure 19). As each component has its Gaussian distribution as a result of how the OMSP code sorts the raw data, therefore, the Gaussian mixing model is heavily simplified because the model no longer needs to identify clusters and sort them into Gaussian distributions.

As each acrylate used to make up a photoresin formulation can be viewed as a cluster within a population of monomers [78], the individual Gaussian fits can be combined following a summation of the methodology [26]. The OMSP code can be made to represent the formulation as a wt% of each component, as the addition of each component OMSP at different wt% causes a modification to the shape of the Gaussian fit until it adopts the desired formulation shape. Therefore, formulation mixing can be computerized, reducing the need for the physical mixing of materials and saving time and resources.

In doing so, the influence of different components could be studied and decisions made for rapid formulation development without the need to physically develop formulations. As a result, a mathematical model was developed, databasing the OMSP peaks for various oligomers and monomer acrylates, and used to perform Gaussian mixing.

Various formulations were made experimentally, and the OMSP was taken for each one. The coefficient of determination (R^2^) was used to analyze the Gaussian mixed samples to the formulation made to judge how well the fit was to the experimental data (Table 11, “Gaussian Mix R^2^”). The same formulations were created through the code, and the peaks were compared through the coefficient of determination (R^2^). The results shown in Table 11 are a very close fit to the experimental results for every formulation considered. The R^2^ error found between the experimental data and the Gaussian mixed data ranged from 0.8433–0.9964. The worst fits in the dataset came from the methacrylate-containing formulations, where inaccuracies in OMSP were already identified with this functional group type. With the exclusion of the methacrylate-containing data, the R^2^ ranged from 0.9406–0.9964 amongst the remaining 20 formulations. The results of this study presented the potential for the computerization of formulation development and the potential to be a key parameter in the use of artificial intelligence in VPP.

### 3.7. Summary of OMSP’s Capabilities

OMSP is a characterization tool that uses ATR-FTIR to analyze the 810 cm^−1^ peak and provide unique quantitative characteristics to acrylates to describe their potential reactivity. OMSP uses a simple graphical and tabular output to summarize the complex chemistry behind various acrylate systems. Due to the fact that each acrylate material has a Gaussian curve that defines its reactive state, it is possible to store the Gaussian curves in a database, allowing for the data to be recalled and used to simulate the mixing of acrylates with high similarity to real-life results. This innovation has provided more clarity in the design and decision-making process surrounding VPP and photoresin design, providing the following benefits and opportunities for advancement for researchers, developers, and manufacturers of acrylate-related works:

Researchers: A clear evaluation of formulations can be made in the literature to indicate how the changes to the acrylate system being studied really influence the results, allowing for the development of photoresin formulations with more confidence. The availability of quantitative data will allow researchers to directly compare their formulations across literature in a less subjective way. Simulation of formulations can be performed in comparison to time consuming manual mixing, allowing for more variations to be added to the literature via simulation and combined with physical experimentation to provide more information and data in ways not previously feasible. Researchers and developers can also benefit from the high precision of OMSP for use as input parameters for artificial intelligence.

Manufacturers: OMSP data can be released with acrylate manufacturer product lines, providing the detail customers desire without having to divulge photoresin compositions, protecting the manufacturer’s intellectual property yet satisfying the needs of the customers. OMSP can be used to evaluate existing product lines and assess gaps in performance for the potential to develop novel acrylates and tailor product lines to better suit application and performance requirements.

OMSP changes the way in which monomers are consumed and polymers are produced, providing a waste minimization route to formulation design in line with the World Health Organization’s Sustainable Development Goal 12: responsible consumption and production [79].

### 3.8. Future Developments

OMSP has shown its capabilities and value in the development and characterization of photoresin components and formulations. OMSP is currently limited solely to the crosslinking capabilities of acrylate materials, but this can be expanded with future studies, including the development of a methacrylate system and the identification of additional functional groups that present other material behavior, such as amines groups causing flexibility.

This study explored the reactivity of formulation when subjected to a fixed exposure intensity and time. In future studies, OMSP will be used to compare responses of formulations to varied exposure intensity and exposure times to detect trends in kinetic behavior.

With further research, OMSP can become an extensive database of UV-curable material reactivity data, allowing for the full digitalization of photoresin development. OMSP’s place in innovative technologies can make it possible for it to be used as a parameter to aid the prediction of ideal formulation performance with the use of artificial intelligence. Additionally, with the use of algorithms such as the Levenberg–Marquardt algorithm and additional parameters, OMSP can be used to break down photoresin formulations into their components, aiding researchers in understanding and identifying photoresin systems as well as component substitutions that yield the same formulation results.

## 4. Conclusions

OMSP is an exciting, novel analysis tool for acrylate characterization, which provides valuable information on the influence components have on the formulation, hence helping scientists and engineers to design the right photoresin for their specific applications and needs. OMSP is based on the analysis of the 810 cm^−1^ peak to determine the peak position and assign it to acrylate functionality to both monomers and mixed acrylate formulations (pseudo-functionality), aspect ratio to assign the distribution of reactivity within an acrylate sample, and peak area to determine the density of alkene bonds in the sample.

This method is currently limited to acrylates, but with further research, the possibilities of OMSP can be expanded. The experiments undertaken in this study have shown a consistent trend in OMSP’s ability to characterize various acrylate monomers, oligomers, and photoresin formulations. The scope of OMSP is currently limited to acrylates; however, there is potential for the further development of OMSP to be applicable to a range of photo-polymerizable photoresin components. The following conclusions were drawn from the study:

1. Acrylate functionality can be linked to unique wavenumbers: The study of various functionality monomers showed that not all acrylates have the same reactivity. The higher the functionality of the acrylates, the lower the vibrational energy attributed to π bond delocalization. In doing so, a peak position could be assigned to different functionalities, making it possible for photoresin formulations to be assigned a pseudo-functionality.
2. The abundance of alkenes for polymerization: Through the assessment of the peak area under the spectral peak used to identify alkene bonds specific to acrylate functional groups, the concentration of alkene bonds available for polymerization could be identified. The method could detect the impact of molecular complexity and voids caused by non-acrylate components, giving insight when studying the impact of filler on the conversion capabilities of a mixture.
3. Shift in reactivity: The study was able to show how the addition of additives, such as dispersants of high acid value, impacted the reactivity of the photoresin through the shift in peak position and the subsequent increase in polymerization, validated through a conversion study.
4. Computerized Mixing: The application of Gaussian mixing to this area of study was successful, allowing for the computerized mixing of acrylate formulations, showing a good likeness to experimentally mixed formulations with an average R^2^ of 0.9673 standard deviations of 0.04342), for all formulations, including ones containing methacrylates and non-acrylate reactive monomers.

## Figures and Tables

**Table 1:** Table of (meth) acrylates used in this study.

Material Sourced from Rahn	Identifier	Functionality	Viscosity (mPa·s)	Molecular Weight (g/mol)
Dipentaerythritol Hexaacrylate	DPHA	6	7000	578
Trimethylolpropane triacrylate	TMPTA	3	110	296
Trimethylolpropane trimethacrylate	TMPTMA	3	55	338
Aliphatic Urethane Acrylate	G-4259	2	11,000	-
Aliphatic Urethane Acrylate	G-4293	2	25,000	-
Amine Acrylate	G-5271	2	1200	-
Epoxy Acrylate	G-2263	2	30,000	-
Aliphatic Urethane Methacrylate	Primary	2	10,000	-
Dipropylene Glycol Diacrylate	DPGDA	2	15	242
1,6-Hexanediol diacrylate	HDDA	2	10	226
Neopentyl Glycol Propoxylate Diacrylate	NPG(PO)2DA	2	15	328
Polyethylene Glycol(200) Diacrylate	PEG(200)DA	2	25	308
Polyethylene Glycol(300) Diacrylate	PEG(300)DA	2	50	408
Isobornyl Methacrylate	IBOMA	1	8	222
Isobornyl acrylate	IBOA	1	8	208
4-Acryloylmorpholine	ACMO	1	12	141
Cyclic Trimethylolpropane Formal Acrylate	CTFA	1	15	200

**Table 2:** Table of formulations.

ID	UA	Primary	AA	EA	DPHA	HDDA	DPGDA	NPG(PO)2DA	IBOMA	IBOA	CTFA	ACMO	IDA	PEG200DA	PEG300DA	TMPTA	TMPTMA	BYK 111
M.1				0.5										0.3		0.2		
M.2				0.5										0.3			0.2	
M.3	0.4				0.1					0.5								
M.4	0.4				0.1				0.5									
C.1	0.8										0.1							
C.2	0.7										0.2							
C.3	0.6										0.3							
C.4	0.6										0.4							
C.5	0.5										0.5							
C.6	0.8											0.1						
C.7	0.7											0.2						
C.8	0.6											0.3						
C.9	0.6											0.4						
C.10	0.5											0.5						
A.1	0.4				0.1		0.5											
A.2	0.4				0.1			0.5										
B.1	0.5				0.1	0.4												
B.2				0.5	0.1	0.4												
B.3			0.5		0.1	0.4												
D.1	0.5													0.1		0.4		
D.2	0.5													0.2		0.3		
D.3	0.5													0.3		0.2		
D.4	0.5													0.4		0.1		
E.1	0.5				0.1	0.4												1
E.2	0.5				0.1	0.4												3
E.3	0.5				0.1	0.4												5
F.1					0.9								0.1					
F.2					0.8								0.2					
F.3					0.64								0.36					
F.4					0.56								0.44					
F.5					0.50								0.50					
F.6					0.45								0.55					
F.7					0.41								0.59					
F.8					0.29								0.71					
F.9					0.20								0.80					

**Table 3:** Defined OMSP code outputs.

Parameter	Definition
File Name	The same name given to the imported file is stored and displayed to assign results to a specific dataset.
Fit Type	Displays whether Gaussian, Lorentzian, or the Linear Combination Pseudo-Voigt method was found to be the best fit.
AIC	The Akaike Information Criterion output for the selected fit type. A larger negative number indicates a better fit.
RMSE	The root mean square error is determined for the selected fit type. The smaller the value, the better the fit.
Peak Position	Shows where the sample’s maximum amplitude is located on the spectrum. This can indicate the reactivity of the sample. A higher degree of red spectral shift is an indication of increased reactivity due to changes in the molecular structure/intermolecular forces influencing the energy level of the alkene bond of interest.
OMSP Area	The normalized peak area of the sample indicates the density of alkene bonds in a particular sample.
Pseudo-Functionality	A functionality that is given to photoresin formulations based on their peak position despite being a multifunctionality mixture.
ΨOMSP Area	The peak area is determined by using the pseudo-functionality home position to calculate the density of alkene bonds in a sample available for photopolymerization.
Aspect Ratio	An insightful parameter that displays information on whether the OMSP output is of a tall or narrow shape, indicating many acrylates in the system vibrating at the same energy level or a shorter, broader peak, suggesting that there is a wide spread of acrylates.

**Table 4:** Acrylate molecules selected to represent the mean energy state for each functionality acrylate.

Functionality	Chemical Name
1	Iso-Decyl acrylate (IDA)
2	1,6-Hexanediol Diacrylate (HDDA)
3	Trimethylolpropane Triacrylate (TMPTA)
4	Pentaerythritol Tetraacrylate (PETA)
5	Dipentaerythritol Pentaacrylate (DPPA)
6	Dipentaerythritol Hexaacrylate (DPHA)

**Table 5:** OMSP functionality home acrylate parameters.

Functionality	Peak Position (cm^−1^)	σ	Aspect Ratio
1	809.81	0.004	203.15
2	808.79	0.0024	39.927
3	807.77	0.0024	23.254
6	805.73	0.0016	4.3711

**Table 6:** Printed specimen results of methacrylate monomers compared to acrylate monomers.

ID	Viscosity (mPa·s)	Conversion (%)	σ	UTS (MPa)	σ	E	σ	Elongation (%)	σ	Hardness	σ
M.1	88	47.10	1.14	40.71	1.04	408.24	8.48	17.12	0.89	83.80	1.25
M.2	173	37.13	3.48	36.46	1.11	393.65	16.99	17.07	0.58	81.54	0.75
M.3	46	24.17	0.67	284.83	13.52	20.86	2.90	74.88	0.85	24.17	0.67
M.4	133	22.17	1.76	222.92	24.98	22.14	0.63	70.70	2.54	22.17	1.76

**Table 7:** Printed specimen results for DPGDA and NPG(PO)2DA differentiation.

ID	Viscosity (mPa·s)	Conversion (%)	σ	UTS (MPa)	σ	E	σ	Elongation (%)	σ	Hardness	σ
A.1	88.00	47.10	1.14	40.71	1.04	408.24	8.48	17.12	0.89	83.80	1.25
A.2	173.00	37.13	3.48	36.46	1.11	393.65	16.99	17.07	0.58	81.54	0.75

**Table 8:** Printed specimen results of formulations containing oligomers of different flexibilities.

ID	Conversion (%)	σ	UTS (MPa)	σ	E	σ	Elongation (%)	σ	Hardness	σ
B.1	22.55	4.57	35.92	4.85	342.59	41.67	15.20	0.81	78.64	3.02
B.2	59.32	4.75	37.51	2.44	391.49	12.29	13.92	1.99	82.20	4.97
B.3	16.11	2.04	3.56	0.52	56.20	7.31	8.69	0.88	54.84	1.99

**Table 9:** Printed specimen results of comparing formulations of different TMPTA concentrations.

ID	UTS (MPa)	σ	E	σ	Elongation (%)	σ	Hardness	σ
D.1	35.56	1.90	338.12	18.76	16.36	1.67	82.60	0.89
D.2	39.47	3.19	373.61	32.72	18.57	0.68	79.80	1.64
D.3	44.45	2.84	441.59	30.13	18.23	1.33	80.70	2.13
D.4	30.45	0.82	303.29	9.56	17.36	0.70	81.80	0.45

**Table 10:** Impact of dispersant amount on the peak position and conversion of the photoresin.

Dispersant Amount	Peak Position	Conversion
1	808.91	40.18
3	808.93	52.71
5	809.70	57.896

**Table 11:** Average OMSP output of formulations in this study.

ID	Gaussian Mix R^2^	OMSP Area	σ	Peak Position	σ	Aspect Ratio	σ	Pseudo-Functionality	Pseudo Area	σ
A.1	0.9882	0.1435	0.0028	808.4785	0.0263	74.5632	0.9766	2.0	0.0776	0.0012
A.2	0.9406	0.1354	0.0001	808.7328	0.0066	77.7593	0.1531	2.0	0.0737	0.0002
B.1	0.9933	0.1408	0.0045	808.9115	0.0793	41.9378	0.6693	2.0	0.0811	0.0022
B.2	0.9901	0.1796	0.0007	807.9112	0.0153	26.1247	0.0376	3.0	0.1355	0.0006
B.3	0.9848	0.1616	0.0001	809.5891	0.0002	85.8764	0.0564	1.0	0.0992	0.0001
C.1	0.9926	0.0853	0.0001	808.9715	0.0040	19.0611	0.0426	2.0	0.0474	0.0001
C.2	0.9932	0.0944	0.0002	808.9818	0.0066	55.4130	0.1660	2.0	0.0524	0.0001
C.3	0.9943	0.1048	0.0002	809.0262	0.0061	100.2605	0.8498	2.0	0.0584	0.0001
C.4	0.9955	0.1119	0.0002	809.0477	0.0077	140.3469	6.1436	2.0	0.0626	0.0001
C.5	0.9963	0.1206	0.0002	809.0362	0.0081	94.6027	121.0432	2.0	0.0674	0.0002
C.6	0.9750	0.0712	0.0001	809.2512	0.0100	112.4926	123.6563	2.0	0.0391	0.0001
C.7	0.9538	0.0690	0.0002	809.5008	0.0016	148.9019	126.0531	1.0	0.0386	0.0002
C.8	0.9152	0.0683	0.0001	809.7751	0.0097	236.3868	24.9293	1.0	0.0393	0.0000
C.9	0.8231	0.0827	0.0006	809.7850	0.0100	110.0589	16.7545	1.0	0.0470	0.0004
C.10	0.8803	0.0647	0.0002	810.0170	0.0071	248.5165	317.9802	1.0	0.0381	0.0002
D.1	0.9963	0.1266	0.0024	808.0475	0.0238	43.3092	45.8575	3.0	0.0955	0.0017
D.2	0.9964	0.1244	0.0042	808.1450	0.0231	48.5235	25.9756	3.0	0.0946	0.0033
D.3	0.9933	0.1174	0.0012	808.3110	0.0379	75.0845	16.1846	2.5	0.0761	0.0154
D.4	0.9943	0.1164	0.0002	808.4077	0.0975	91.6005	4.6589	2.0	0.0683	0.0055
E.1		0.1349	0.0004	808.9072	0.0086	43.4779	0.0296	2.0	0.0777	0.0004
E.2		0.1337	0.0001	808.9324	0.0023	43.9323	0.0099	2.0	0.0771	0.0001
E.3		0.0945	0.0019	809.6979	0.0151	30.0811	14.6260	1.0	0.0579	0.0011
F.1	0.99133	0.1698	0.0002	806.5943	0.0028	4.6328	0.5158	5.0	0.1479	0.0003
F.2	0.97242	0.1812	0.0004	807.1414	0.0025	18.5927	18.7931	4.0	0.1318	0.0003
F.3	0.97845	0.1860	0.0001	807.4527	0.0002	20.7377	12.5333	3.0	0.1371	0.0002
F.4	0.97376	0.1893	0.0003	807.6646	0.0016	32.6495	0.0931	3.0	0.1407	0.0001
F.5	0.96759	0.1933	0.0011	807.8435	0.0037	35.2491	0.0228	3.0	0.1447	0.0007
F.6	0.96651	0.1943	0.0001	808.0486	0.0165	55.0680	26.1248	3.0	0.1470	0.0001
F.7	0.96648	0.1955	0.0012	808.1543	0.0023	15.4292	0.1092	3.0	0.1486	0.0009
F.8	0.96725	0.1973	0.0002	808.5364	0.0049	35.6670	25.1628	2.0	0.1043	0.0000
F.9	0.97403	0.1990	0.0004	808.8632	0.0069	49.4474	41.8887	2.0	0.1084	0.0002
M.1	0.9958	0.1663	0.0002	807.2729	0.0022	28.0230	0.0205	3.0	0.1202	0.0001
M.2	0.9682	0.1784	0.0033	807.6898	0.0436	113.8961	18.4272	3.0	0.1321	0.0021
M.3	0.9931	0.1248	0.0008	808.7009	0.0028	87.9753	4.2273	2.0	0.0674	0.0004
M.4	0.8433	0.1244	0.0004	809.1787	0.0079	128.7159	7.0550	2.0	0.0720	0.0003

## Data Availability

The original contributions presented in this study are included in the article. Further inquiries can be directed to the corresponding author.
